# HAIC as a potential therapy for esophageal cancer patients with liver metastasis: a retrospective cohort study

**DOI:** 10.3389/fmed.2023.1143617

**Published:** 2023-05-05

**Authors:** Fengxiao Dong, Guang Cao, Zhihao Lu

**Affiliations:** ^1^Department of Gastrointestinal Oncology, Peking University Cancer Hospital, Key Laboratory of Carcinogenesis and Translational Research (Ministry of Education), Peking University Cancer Hospital and Institute, Beijing, China; ^2^Department of Interventional Therapy, Peking University Cancer Hospital, Key Laboratory of Carcinogenesis and Translational Research (Ministry of Education), Peking University Cancer Hospital and Institute, Beijing, China

**Keywords:** HAIC, local therapy, liver metastasis, gastrointestinal tumor, esophagus squamous cell carcinoma

## Abstract

**Methods:**

This was a single-arm historical cohort study of ESCC patients with synchronous or heterochronous LM between December 2014 and July 2021 at the Department of Gastrointestinal Oncology. The patients were treated with HAIC for LM, and regular image assessments were performed according to the judgment of the interventional physician. Liver progression-free survival (PFS), liver objective response rate (ORR), liver disease control rate (DCR), overall survival (OS), adverse events (AEs), treatment information, and basic characteristics were observed retrospectively.

**Results:**

Overall, a total of 33 patients were enrolled in this study. All included patients received catheterized HAIC therapy, with a median of three (ranging from 2 to 6) sessions. The treatment response of liver metastatic lesions included partial response (PR) in 16 (48.5%) patients, stable disease (SD) in 15 (45.5%) patients, and progressive disease (PD) in two (6.1%) patients, for an ORR of 48.5% and a DCR of 93.9%. The median liver PFS was 4.8 months (95% confidence interval (CI): 3.0–6.6 months), and the median OS was 6.4 months (95% CI: 6.1–6.6 months). Patients who achieved PR at the liver metastasis site after HAIC were more likely to have a longer OS than those who achieved SD or PD. Grade 3 AEs occurred in 12 patients. The most common grade 3 AE was nausea, occurring in 10 (30.0%) patients, followed by abdominal pain in three (9.1%) patients. Only one patient showed grade 3 elevation of alanine aminotransferase (ALT)/aspartate aminotransferase (AST), and one patient suffered from grade 3 embolism syndrome AEs. Grade 4 adverse events, followed by abdominal pain, occurred in one patient.

**Conclusion:**

Hepatic arterial infusion chemotherapy might be an option as a regional therapy for ESCC patients with LM, as it is acceptable and tolerable.

## Background/Introduction

Esophageal cancer (EC) ranks seventh in morbidity (604,000 new cases) and sixth in mortality (544,000 deaths) among cancer deaths, the latter signifying that esophageal cancer was responsible for one in every 18 cancer deaths in 2020 (1). More than half of the burden is in China, with the predominant subtype being esophageal squamous cell cancer (ESCC). Most ESCC patients, approximately over 50%, are diagnosed at an advanced stage, with spread to distant organs or lymph nodes or to nearby organs and lymph nodes, and mainly require systematic therapy, such as radical/definitive concurrent radiochemotherapy (CRT) and chemotherapy with or without immunotherapy, which have an unsatisfactory 5-year survival rate of ~10%. Patients with distant metastatic lesions have a worse prognosis. Several studies on the patterns of distant organ metastases in EC from the Surveillance, Epidemiology, and End Results (SEER) program showed that the liver was the most common metastatic site in EC, followed by the lung, bone, and brain (2). Other studies reported similar results: distant liver metastasis (32.4%), followed by distant lymph nodes (28.4%), lung (20.5%), bone (15.3%), and brain (3.4%), were the common metastasis sites (3, 4). Regarding the different histological subtypes, esophageal adenocarcinoma (EAC) is more likely to metastasize to the brain and liver and less likely to metastasize to the lung, and the ESCC and EAC subtypes showed no difference in metastasis to distant lymph nodes or bone (3). It is not uncommon that some EC patients suffer from synchronous or heterochronic liver metastasis (LM).

Approximately 60% of patients with colorectal cancer (CRC) develop LM during the course of their disease (5). Hepatic arterial infusion chemotherapy (HAIC), which delivers high drug concentrations to the tumor but results in less systemic toxicity, has been widely employed for the treatment of LM in CRC (6) when systemic chemotherapy fails (7–9). However, the efficacy and safety data of HAIC using oxaliplatin combined with 5-fluorouracil (5-FU) for unresectable LM for ESCC patients are scarce.

In a very early study dating back 20 years ago, Nakajima et al. (10) reported a retrospective analysis of eight patients who underwent hepatic arterial infusion between 1993 and 1998 and showed a 50% overall response rate, with a good quality of life. In our daily practice, we propose that ESCC patients who have a good Eastern Cooperative Oncology Group (ECOG) performance status (PS) score of 0–1 receive HAIC therapy for LM to achieve longer regional disease control. The aim of this study was to provide insight into the efficacy and safety of HAIC in ESCC patients with LM.

## Materials and methods

This was a single-institution, single-arm retrospective cohort study. All consecutive ESCC patients with LM receiving HAIC therapy from December 2014 to July 2021 were included in the study. We assessed tumor response according to the Response Evaluation Criteria in Solid Tumors 1.1 (RECIST1.1), liver progression-free survival (PFS), overall survival (OS), liver objective response rate (ORR), liver disease control rate (DCR), and incidence of severe adverse events (AEs) of HAIC. Basic clinical characteristics and serological test results were collected retrospectively. This study was performed according to the Declaration of Helsinki (11).

### Statistical analysis

The continuous variables with a normal distribution are expressed as means ± standard deviations, and those with a skewed distribution are expressed as medians (range). Categorical variables are expressed as *n* (%). Survival analysis was performed using the Kaplan–Meier method. Statistical analyses were performed with GraphPad Prism statistical software version 8.0 (GraphPad Software) and the Statistical Package for Social Sciences software (version 25.0, SPSS). There was no hypothesis testing.

## Results

### Clinical characteristics

Overall, a total of 33 patients were enrolled in this study. The median age was 63 (ranging from 56 to 68) years, and 30 (90.9%) patients were men. The ECOG performance status was 0 in 20 (60.6%) patients and 1 in 13 (39.4%) patients. Five (15.2%) patients had esophageal lesions located in the upper thoracic region, 10 (30.3%) patients had esophageal lesions located in the middle thoracic region, and 18 (54.5%) patients had esophageal lesions located in the lower thoracic region. All patients had ESCC, with 32 (97.0%) grading as moderately or poorly differentiated. Overall, 13 (39.4%) patients had synchronous LM, 20 (60.6%) patients had heterochronous LM, and 18 (54.5%) patients suffered from extra-hepatic metastasis. Fifteen (45.5%) patients had received previous esophagectomy treatment. Nine (27.3%) patients, 14 (42.4%) patients, and 10 (30.3%) patients received HAIC in the first line, second line, and third line, respectively (see Table 1).

**Table 1:** Basic characteristics.

**Variable**	**Number**	**Percentage (%)**
**Age**		
Median (range)	63	56–68
**Sex**		
Male	30	90.9
Female	3	9.1
**ECOG score**		
0	20	60.6
1	13	39.4
**Cancer location**		
Upper thoracic	5	15.2
Middle thoracic	10	30.3
Lower thoracic	18	54.5
**Pathological subtype**		
Squamous	33	100
**Differentiation grade**		
Highly differentiated	1	3.0
Mildly differentiated	16	48.5
Poorly differentiated	16	48.2
Previous surgery	15	45.5
**Child–Pugh stage**		
Stage A	33	100
Extra-hepatic metastasis	18	54.5
**Hepatic metastasis lesion**		
Synchroneity	13	39.4
Heterochrony	20	60.6
**Treatment line of HAIC**		
First line	9	27.3
Second line	14	42.4
Third line	10	30.3

### Treatment profile of HAIC

All the included patients received catheterized HAIC therapy, with a median of three sessions (ranging from 2 to 6). The HAIC chemotherapy was a platinum-based regime combined with docetaxel and 5-fluorouracil (see Table 2).

**Table 2:** Treatment information.

**Variable**	**Number**	**Percentage (%)**
**HAIC technology**		
Catheterized	33	100
**HAIC cycles**		
2	11	33.3
3	7	21.2
4	9	27.3
5	3	9.1
6	3	9.1
**HAIC regime**		
Cis-platinum-based^*^	33	100

* Cisplatinum + 5-FU + docetaxel.

### Tumor response and safety of HAIC

The treatment responses of liver metastatic lesions were partial response (PR) in 16 (48.5%) patients, stable disease (SD) in 15 (45.5%) patients, and progressive disease (PD) in 2 (6.1%) patients, for an ORR of 48.5% and a DCR of 93.9% (see Table 3). The median liver PFS was 4.8 months (95% CI 3.0–6.6 months), and

**Table 3:** Treatment response and adverse events.

**Variable**	**Number**	**Percentage (%)**
**Therapy response**		
PR	16	48.5
SD	15	45.5
PD	2	6.1
**Grade 3 AEs**		
Nausea	10	30.3
ALT/AST elevation	1	3.0
Abdominal pain	3	9.1
Embolism syndrome	1	3.0
**Grade 4 AEs**		
Abdominal pain	1	3.0

the median OS was 6.4 months (95% CI 6.1–6.6 months; see Figure 1). Patients who achieved PR in LM after HAIC were more likely to have a longer OS than patients with SD or PD (see Figure 2). Both the liver PFS and OS had no relationship with whether the metastasis site was synchronous or heterochronous (see Figure 3). Moreover, we did not observe a difference in the liver PFS and OS between the extra-hepatic metastasis group and the non-extra-hepatic metastasis group (see Figure 4). We show the CT images of one patient from the cohort (see Figures 5A–D) to illustrate the therapeutic effect. This patient suffered liver progression when they received immunotherapy plus anti-angiogenesis as the third line, with exposure to nedaplatin and paclitaxel in previous therapy. Before HAIC, the LM occupied almost two-thirds of the volume of the whole liver tissue and caused painful distension in the right upper quadrant. After two cycles of HAIC, the tumor regressed greatly, resulting in a partial response in terms of the local evaluation.

**Figure 1:**
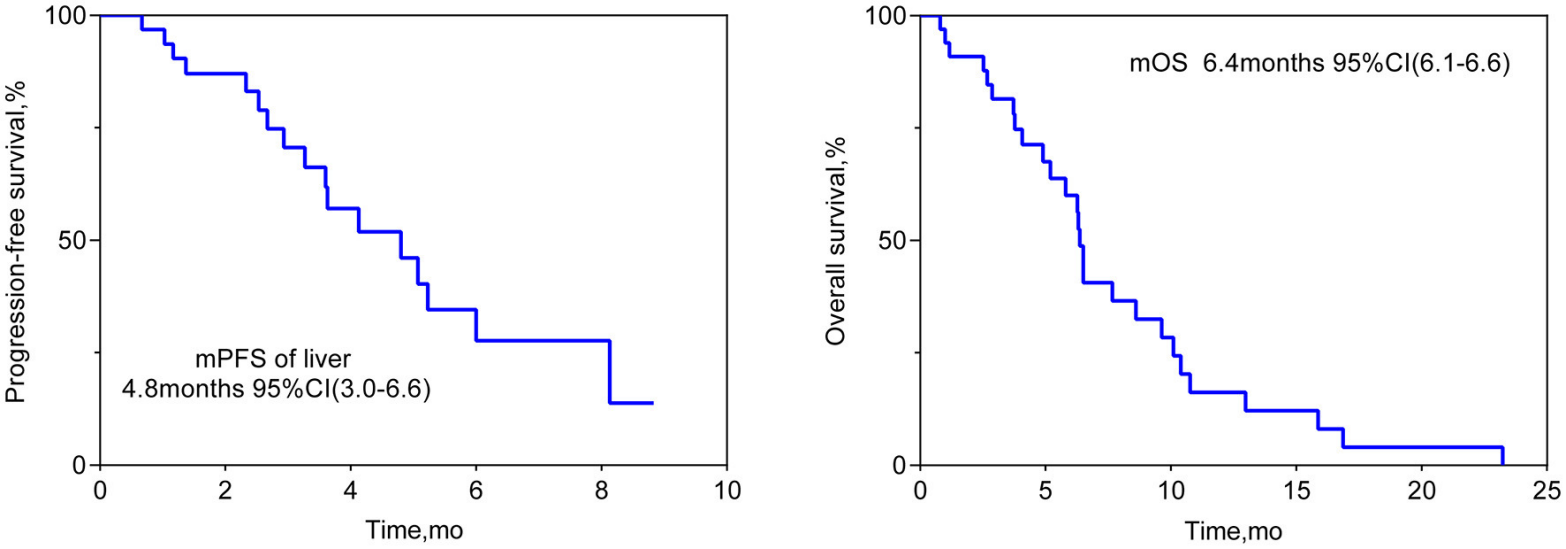
Liver progression-free survival and overall survival.

**Figure 2:**
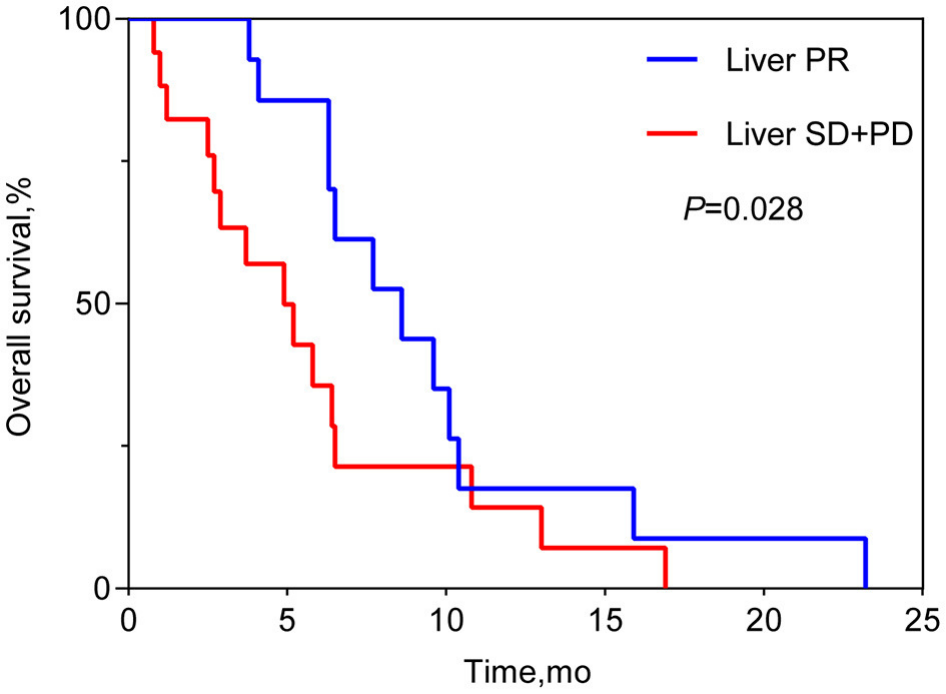
Overall survival between the PR group and the SD+PD group.

**Figure 3:**
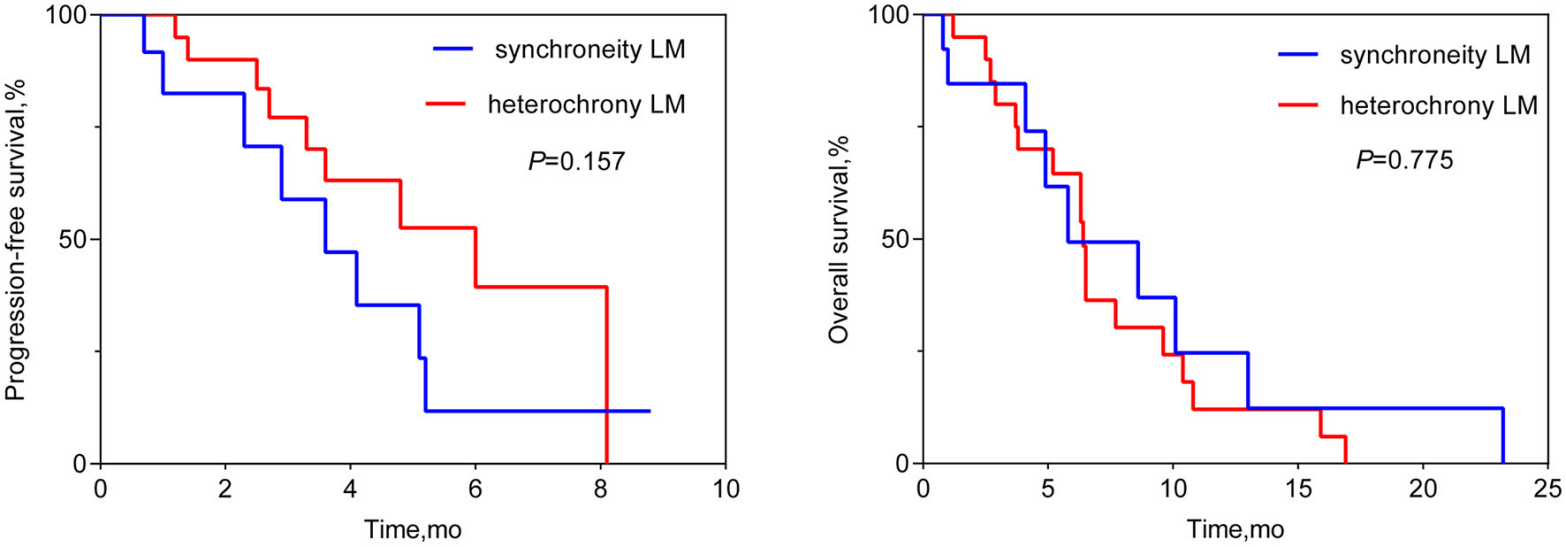
Liver progression-free survival and overall survival between the synchronous LM group and the heterochronous LM group.

**Figure 4:**
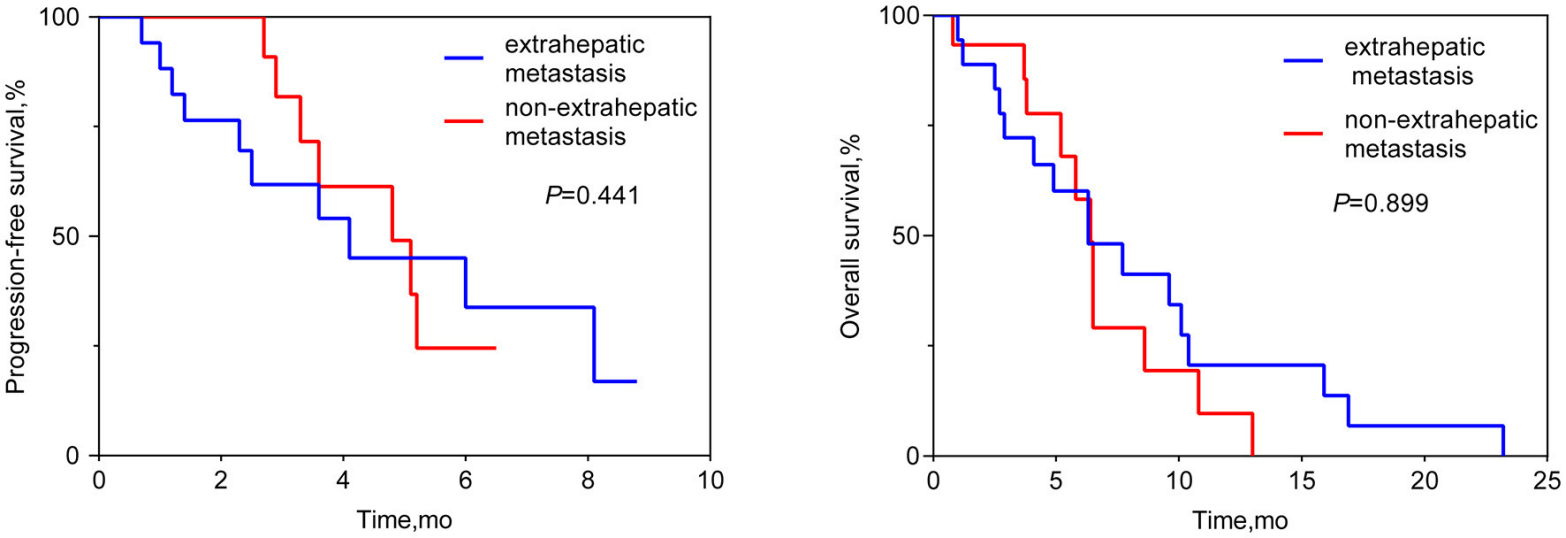
Liver progression-free survival and overall survival between the extra-hepatic metastasis subgroup and the non-extra-hepatic metastasis subgroup.

**Figure 5:**
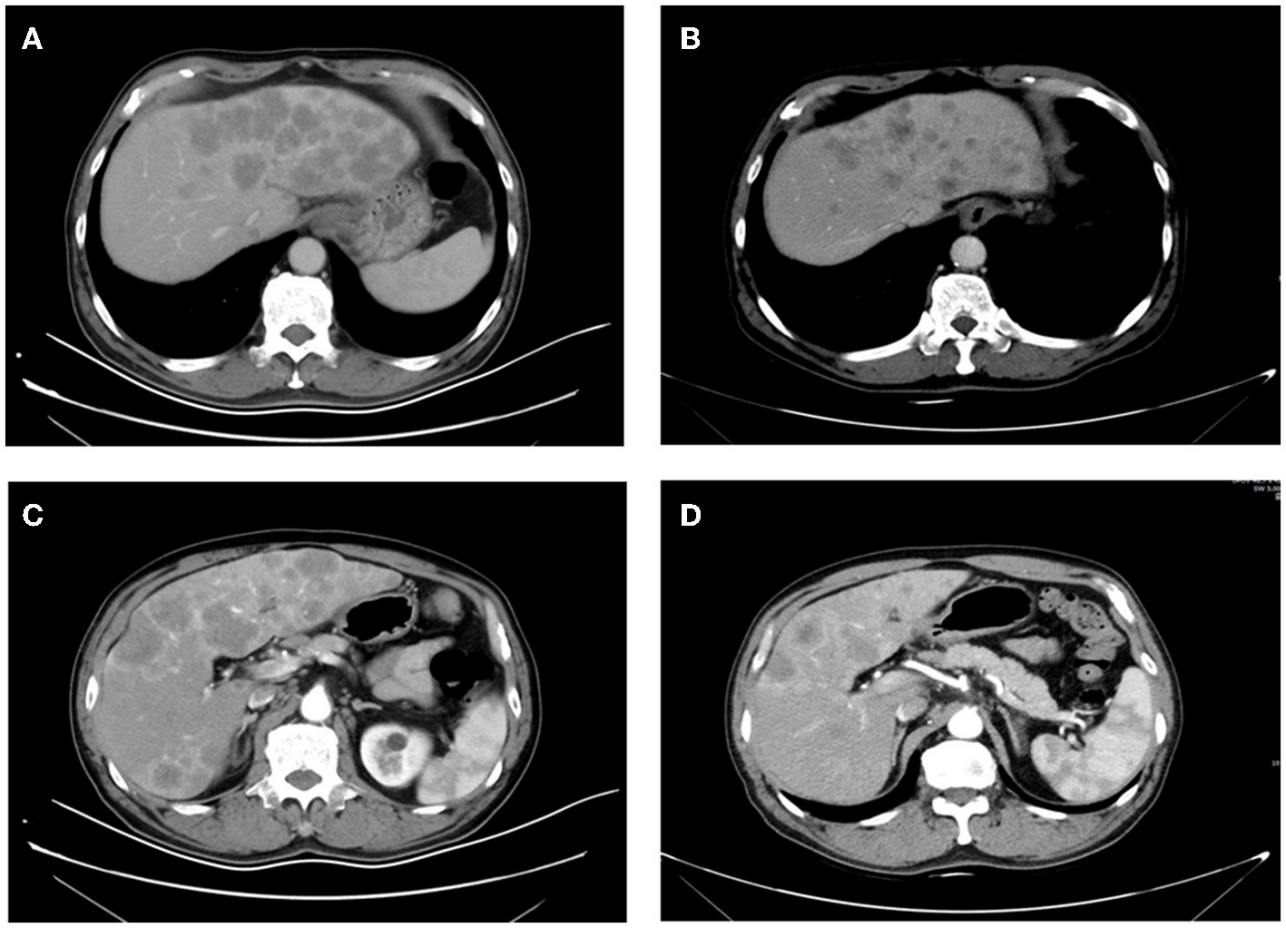
Images **(A, B)** were taken before HAIC, and **(C, D)** were taken after two cycles of HAIC.

Grade 3 AEs occurred in 12 patients. The most common grade 3 AE was nausea, in 10 (30.3%) patients, followed by abdominal pain in three (9.1%) patients. One patient showed grade 3 elevation of ALT/AST, and one patient suffered from grade 3 embolism syndrome. A grade 4 AE, abdominal pain, occurred in one patient (see Table 3).

## Discussion

In this cohort study, we retrospectively reviewed a cohort of 33 ESCC patients with LM receiving HAIC for regional metastasis and described the liver ORR, liver DCR, liver PFS, OS, and AEs of the treatment population. The liver ORR was 48.5%, and the liver DCR was 93.9%, achieving a median liver PFS of 4.8 months (95% CI: 3.0–6.6 months) and a median OS of 6.4 months (95% CI: 6.1–6.6 months). This study revealed that HAIC for LM in ESCC patients is effective and feasible.

There is no robust evidence supporting the application of HAIC in ESCC patients with LM, with not only a lack approved guidelines but also the exact regimen. LM is also a major cause of mortality in CRC (12) and occurs in approximately 40% of CRC patients during the course of the disease, either synchronously (20%) or metachronously (20%) (13, 14). HAIC therapy can achieve a more than 5-fold increase in drug concentration within the liver compared to that achieved through the intravenous route (15, 16), resulting in a 2- to 3-fold increase in the response rate when using 5-FU and oxaliplatin (17). HAIC has been developed to increase the local concentration of cytotoxic agents for treating LM and, therefore, to improve hepatic disease control and colorectal liver metastasis (CRLM) resectability (18). Thus, we drew on the experience from the treatment of CRLM. In our study, all patients were mainly treated with a platinum-based regimen combined with 5-FU: cisplatin 75 mg/m2 d1 2h, docetaxel 85 mg/m^2^ d1 2h, and 5-fluorouracil 800 mg/m^2^ 20 h on days 1–2, q3-4w.

In this retrospective cohort study, a total of 33 ESCC patients with LM received HAIC for liver metastatic lesions. The liver ORR was 48.5%, and the liver DCR was 93.9%, achieving a median liver PFS of 4.8 months (95% CI: 3.0–6.6 months) and a median OS of 6.4 months (95% CI: 6.1–6.6 months). All the patients included had the most tumor burden in the liver metastasis, predicting a worse outcome. The data from the Surveillance, Epidemiology, and End Results (SEER) database showed that the median OS for ESCC patients with liver metastases was only 5 months (19). In our cohort, nine (27.3%) patients, 14 (42.4%) patients, and 10 (30.3%) patients received HAIC in the first line, second line, and third line, respectively. According to previous reports, second-line chemotherapy of ESCC only had an ORR of ~6.4–9.8% (20–22), which is lower than that of our HAIC cohort (48.5%). HAIC could increase the local control and achieve a median overall survival of 6.4 months, which is longer than that in the control cohort in the previous studies (20–22). In total, 63.6% (21/33) of patients suffered progression in the liver metastasis site when receiving the systemic treatment. This benefit to liver PFS achieved by HAIC therapy is critically important for advanced ESCC patients. According to the subgroup analysis, the difference in OS can be seen between the subgroup of PR and the subgroup of SD or PD, while there was no difference in OS neither between the extra-hepatic metastasis subgroup and the non-extra-hepatic metastasis subgroup nor between the synchronous hepatic metastasis subgroup and the heterochronous hepatic metastasis subgroup. After the failure of systemic therapy in LMs, HAIC intervention treatment can be beneficial for attaining local control of LMs. Hopefully, regional control of LMs can prolong the survival of advanced ESCC patients. Thus, we must make every effort to control liver metastasis sites to the fullest. Simply, our study wants to show that HAIC treatment for local liver lesions is feasible and could offer regional control to provide insight for future clinical trials.

Recently, immunotherapy for ESCC has achieved landmark progress (23), and immunotherapy together with anti-vascular agents has become the standard therapy for hepatocellular carcinoma (HCC). We suppose that HAIC combined with systematic immunotherapy with or without anti-vascular drugs may yield a longer hepatic PFS and a better condition for ESCC patients with LM. One prospective trial is ongoing in our center to explore the efficacy of HAIC combined with immunotherapy for ESCC patients with LM, the results of which are highly anticipated.

As a single-arm retrospective cohort study, this study had some limitations: (1) the retrospective nature of the study and the sample size was too small to conduct a stratification analysis to detect potential prognosis-related risk factors. (2) When deciding whether to receive HAIC, there was a selection bias between patients with ECOG 0-1 and patients with scores over 1. (3) There was no control group to include in the comparison, and future prospective studies are needed.

## Conclusion

Hepatic arterial infusion chemotherapy might become a treatment strategy for ESCC patients with liver metastasis as it is feasiable and durable. Future prospective studies are needed to verify the efficacy of HAIC for ESCC with liver metastasis.

## Data availability statement

The original contributions presented in the study are included in the article/Supplementary material, further inquiries can be directed to the corresponding author/s.

## Ethics statement

Written informed consent was not obtained from the individual(s) for the publication of any potentially identifiable images or data included in this article.

## Author contributions

ZL and GC were responsible for conceptualization and interpretation, reviewed, and edited the manuscript. GC, ZL, and FD were responsible for methodology. GC and FD carried out the formal analysis. FD wrote the original draft. ZL were responsible for supervision. All authors contributed to the article and approved the submitted version.
